# Variation of Residual Sexuality Rates along Reproductive Development in Apomictic Tetraploids of *Paspalum*

**DOI:** 10.3390/plants11131639

**Published:** 2022-06-21

**Authors:** Anna Verena Reutemann, Ana Isabel Honfi, Piyal Karunarathne, Fabiana Eckers, Diego Hernan Hojsgaard, Eric Javier Martínez

**Affiliations:** 1 Instituto de Botánica del Nordeste (IBONE-UNNE-CONICET), Facultad de Ciencias Agrarias, Universidad Nacional del Nordeste (FCA-UNNE), Corrientes 3400, Argentina; 2 Programa de Estudios Florísticos y Genética Vegetal, Instituto de Biología Subtropical (PEFyGV, IBS-UNaM-CONICET), Posadas 3300, Argentina; ahonfi@gmail.com (A.I.H.); faby_eckers@hotmail.com (F.E.); 3 Department of Systematics, Biodiversity and Evolution of Plants, Albrecht-von-Haller Institute for Plant Sciences, University of Goettingen, 37073 Goettingen, Germany; piyal.karunarathne@ebc.uu.se (P.K.); hojsgaard@ipk-gatersleben.de (D.H.H.); 4 Evolutionary Biology Center, Uppsala University, 752 36 Uppsala, Sweden

**Keywords:** apospory, cytoembryology, facultative apomixis, flow cytometry, *Paspalum cromyorhizon*, *P. maculosum*, polyploidy, progeny test, residual sexuality

## Abstract

Most apomictic plants are facultative, maintaining the ability to reproduce sexually at different frequencies depending on the taxa, ploidy, and reproductive stage. In this context, *Paspalum* species are good model systems for studies evaluating the varying levels of apomixis expression. We aimed to identify, in apomictic tetraploid *Paspalum* species, the degree of apomixis and residual sexuality in three stages of reproductive development, and if their expression varies along them in order to predict their realized impact on the genetic diversity of future generations. Three main stages in the reproductive development (i.e., ovule, seed, and progeny) were studied in tetraploids from populations of *P. cromyorhizon* and *P. maculosum*. Mature ovules were studied using cytoembryological analysis, seeds by flow cytometry, and progeny tests with molecular markers. The expression of sexuality and apomixis was compared in each stage. We observed a decline in expression of sexual reproduction through the consecutive stages, jointly with an increase of apomixis expression. Both species showed at least one tetraploid plant capable of producing progeny by sexual means. These small rates of sexually originated progeny prove the ability of apomictic plants to produce low levels of genetic variation through rare events of sexuality. This study also demonstrates the importance of analyzing different reproductive stages in order to get a whole picture of the reproductive outcomes in plant evolution.

## 1. Introduction

Apomixis refers to asexual propagation by seeds in plants, wherein the progeny are genetically identical to the maternal plant [1]. It is a process composed of three components: apomeiosis (meiosis bypass), parthenogenesis (formation of an embryo without fertilization), and autonomous endosperm development or pseudogamy (fertilization of the polar nuclei without fertilization of the egg cell).

Apomixis is scattered among multiple taxa, showing a polyphyletic origin, and different forms of apomixis: sporophytic (adventitious embryogenesis) and gametophytic apomixis (diplospory, apospory) [2,3]. Gametophytic apomixis in nature is strongly associated with polyploidy and almost all gametophytic apomictic species are polyploids that show an association to sexual relatives, typically diploids [4]. Gametophytic apomixis involves the formation of unreduced embryo sacs from the megaspore mother cell itself after bypassing meiosis (diplospory) or from a nucellar cell of the ovule (apospory) [5,6]. In both cases, the embryo originates by parthenogenesis of the non-reduced egg cell. In addition, the endosperm formation may require the fertilization of polar nuclei (pseudogamy) or autonomous development.

Most apomictic plants are facultative, maintaining the ability to reproduce sexually at different—often low—frequencies depending on the taxa, the environmental conditions, and the ploidy level, as well as the mechanism of apomictic embryo formation [7,8,9,10,11]. This residual sexuality benefits from using a very sophisticated combination of reproductive strategies, generating diversity and, concurrently, allowing the best fitted individuals to propagate clonally [8,12,13]. 

Apomixis is assumed to result from temporal and spatial changes in important stages of sexual development, caused by mutations or epigenetic modifications that arise after hybridization and polyploidization events [2,14,15]. As apomixis and sexuality coexist in the same plant or even in the same ovule [1,4,16], it has been proposed that apomixis emerged from the rearrangement of sexual developmental programs [17,18,19,20]. Contrary to this, other authors indicate that the main molecular components associated with apomixis (apomeiosis and parthenogenesis) may have emerged before sex [21,22]. During the evolution of eukaryotes, these components may have been retained to different levels in some organisms. In this scenario, hybridization, polyploidy, and other environmental factors could trigger apomixis in plants that conserved such components in variable rates (see [21,22,23,24]). In both models, the apomictic and sexual pathways co-exist in facultative apomictic plants, being sexuality strongly repressed or expressed at very low levels under normal conditions. Stress situations can de-repress sexuality and increase the number of sexual embryo sacs in apomictic plants [25]. The coexistence of both reproductive pathways allows us to hypothesize that sexuality can be repressed at variable rates in apomictic plants depending upon the developmental stage of the reproductive pathway at focus. 

From the meiosis to the mature seed phase until offspring establishment, competition occurs between apomictic and sexual pathways. The frequency of facultative sexuality may vary under the influence of pollinators, the timing of pollination, the flowering stage, the origin of pollen grains, or the environmental conditions [23,26,27,28,29]. This competition during the establishment of a new generation usually favors apomixis by suppressing the sexual pathway [2,30,31]. Nevertheless, almost all the studied aposporic apomictic species show some potential for sexuality, which is realized to some extent [24,31,32,33,34,35]. 

Studies with natural apomictic populations are important in order to determine the origin and maintenance of apomixis in these groups and may consequently be useful in plant breeding programs. Moreover, it is essential to clarify the different reproductive strategies adopted by a group for a better understanding of its evolution [36,37]. In this context, *Paspalum* L. can be considered a good model for studies involving facultative apomixis. *Paspalum cromyorhizon* Trin. Ex. Döll and *P. maculosum* Trin. are agamic complexes composed of sexual diploids (2*n* = 2*x* = 20) and facultative apomictic tetraploids (2*n* = 4*x* = 40) [38,39,40]. Previous studies in *Paspalum* species established that genetic or epigenetic background, ploidy levels, as well as environmental factors, influence the observed rates of apomixis and sexuality [23,24,31,41]. On one hand, diploids of *Paspalum* are able to produce aposporous embryo sacs, but seeds are only sexually produced [27,39,42,43,44], although low rates of functional apomixis were recently noticed [45,46]. On the other hand, previous studies in facultative apomictic tetraploids of *Paspalum* proposed that both reproductive pathways coexist at varying rates at the beginning of female development, but only apomixis succeed to produce viable progeny [31]. 

Our objectives were to verify the coexistence of the apomictic and sexual pathways, to determine the degree in which they are expressed in each reproductive stage, and if this expression varies through the reproductive development. We provide evidence of the rates of sexually originated progeny in apomictic *Paspalum* species. We evaluated the ability to form reduced female gametophytes and their functionality in two apomictic tetraploid *Paspalum* species, examining whether both sexuality and apomixis events might coexist at the ovule stage. Furthermore, we analyzed whether the species maintains the expected rates of sexuality along consecutive developmental stages, at ovules, seed, and progeny obtention, and compared the relative expression of functional sex and apomixis throughout the reproductive stages.

## 2. Results

### 2.1. Proportions of Sexual and Aposporic Megagametophytes in Mature Ovules

The reproductive pathways of 27 tetraploid individuals from four natural populations of *P. cromyorhizon* and two natural populations of *P. maculosum* are summarized in Table 1. 

The tetraploids from population M1 of *P. maculosum* showed ovules bearing MES and ovules bearing MES+AES (Table 1). In addition, tetraploids of population M2 also showed ovules bearing only AES (Table 1). The percentage of ovules with IES in this species ranged from 9.6–11.7%. The 4*x* cytotype of *P. maculosum* showed significant differences favoring sexuality in both populations (*p* < 0.001, Table 1), with meiotic pathway dominating at the ovule stage. 

The tetraploids of *P. cromyorhizon* populations showed ovules bearing MES, AES, and MES + AES (Table 1). Tetraploids from population C4 showed the highest values of ovules carrying AES. Ovules with IES in this species ranged from 1.3–18.3%. Tetraploids from *P. cromyorhizon* populations showed similar proportions for both reproductive pathways and there were not significant differences in either population (Table 1). 

Pro-embryos were observed in the AES of mixed ovules in tetraploids of M1 population of *P. maculosum* (6%) and tetraploids of three populations of *P. cromyorhizon* (C2: 7.8%, C3: 8%, C4: 3.9%), evidencing the parthenogenetic development of the egg cell.

### 2.2. Reproductive Origin of Seeds 

The reproductive origin of ca. 800 mature seeds of tetraploids from *P. maculosum* and *P. cromyorhizon* populations is summarized in Table 2. 

The populations of *P. maculosum* showed dissimilar behaviors at the seed level. The tetraploids from population M1 showed similar values of sexual and apomictic seeds and there were not significant differences (Table 2). In contrast, the tetraploids from population M2 showed a significantly higher proportion of apomictic seeds than sexual ones (*p* < 0.001, Table 2). 

In *P. cromyorhizon*, all populations showed a predominance of apomictic seeds and significant difference between both reproductive pathways (*p* < 0.001, Table 2). A few tetraploids from populations C2 and C3 showed seeds with a 2:6 embryo: endosperm DNA content ratio. These seeds were considered to have an apomictic origin because the embryo has a parthenogenetic origin (2*n* + 0), whereas the endosperm comes from the union between the two polar nuclei with either two reduced sperm nuclei ((2*n*:2*n*) + *n*+ *n*) or one spermatic nucleus from an unreduced pollen grain ((2*n*:2*n*) + 2*n*). 

### 2.3. Proportions of Residual Sexuality Assessed by Progeny Tests 

In total, 474 progenies were evaluated using ISSR markers. A total of 153 and 187 ISSR bands were evaluated for *P. maculosum* and *P. cromyorhizon*, respectively. The percentages of polymorphic bands ranged 0.0–38.7% in *P. maculosum* and 0.0–17.1% in *P. cromyorhizon* (Table S2). 

**Figure 1:**
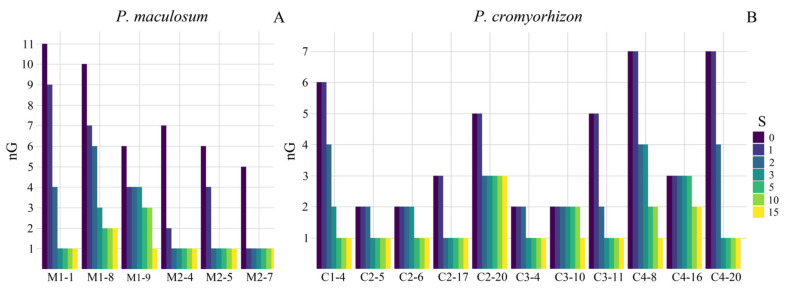
Number of genotypes (nG) considering varying numbers of mutational steps (S). (**A**) Genotype numbers in the progeny of six apomictic genotypes from two populations of *P. maculosum*. (**B**) Genotype numbers in the progeny of 11 apomictic genotypes of four populations of *P. cromyorhizon*. Apomictic genotypes with all clonal progeny considering S = 0 are not presented (C1-20, C2-15, C3-15 and 19, C4-3 and 12). With increasing S, the nG decreased in nearly all the progenies. nG = 1 indicates that all the offspring have the maternal genotype.

All *P. maculosum* populations showed a significantly higher number of clonal than non-clonal progeny (*p* < 0.001, Table 3). The three apomictic genotypes analyzed from population M2 had only clonal progeny (apomictic origin, Table 3 and Table S3). In contrast, only one maternal apomictic genotype (M1-1, Figure 1) from population M1 showed all progeny genotypically identical to the maternal genotype. The remaining two maternal apomictic genotypes (M1-8 and M1-9, Figure 1) showed at least two non-clonal genotypes among descendants (considering S = 3, Figure 1, Table S4). The maternal apomictic genotype M1-9 had three nC progeny (*n* = 20) with genotypes differentiated among them and with the maternal genotype (Table S4). The apomictic genotype M1-8 had three nC progeny (*n* = 20) and two non-clonal genotypes (Table S4). One of these non-clonal genotypes was shared by two progeny and the other non-clonal genotype was observed only in one progeny (Table S4). When increasing the number of mutational steps (S = 0 to 15) the differences among the maternal apomictic genotype and their nC progeny were reduced, but even with S = 10, both maternal apomictic genotypes in M1 showed at least one progeny with a non-clonal genotype (nG ≠ 1, Figure 1). 

In *P. cromyorhizon*, we analyzed the offspring from two maternal apomictic genotypes in C1, and five maternal apomictic genotypes in the remaining three populations (Table 3). All populations showed at least one maternal apomictic genotype with nC progeny (considering S = 3, Table 3 and Table S4). All maternal apomictic genotypes of *P. cromyorhizon* showed a significantly higher number of clonal than non-clonal progeny (*p* < 0.001, Table 3). In population C1, only one maternal apomictic genotype (C1-4, Figure 1) had one progeny that was genotypically different. However, repeating the analysis with S = 5, differences among the maternal genotype and this nC progeny′s genotype disappeared (Figure 1). In the C2 population, two maternal apomictic genotypes (C2-6 and C2-20, Figure 1) had nC progeny. Using S = 10, only the maternal genotype C2-20 maintained its nC progeny (Figure 1). The same pattern was observed in the population C3, which had only one maternal apomictic genotype (C3-10, Figure 1) with two nC progeny (Figure 1). The population C4 had the highest number of nC progeny (Table 3). Only two maternal apomictic genotypes (C4-8 and C4-16, Figure 1) had nC progeny with S = 3. The maternal genotype C4-8 showed three nC progeny (Table 3). Each progeny had a unique genotype (S = 3, Figure 1 and Table S3). When considering S = 15, differences among these genotypes and the maternal apomictic genotype disappeared (Figure 1). The maternal genotype C4-16 had two non-clonal genotypes among its offspring (S = 3, Figure 1). One of these non-clonal genotypes was represented by one descendant, and the other non-clonal genotype was observed in five progenies (Table 3 and Table S3). When considering S = 10, these two non-clonal genotypes were reduced to one, but still differed from the maternal apomictic genotype (Figure 1). 

These results show that some tetraploids from *P. maculosum* and *P. cromyorhizon* have the capability to originate sexual progeny at low frequencies (*P. maculosum*: 0–10%, *P. cromyorhizon:* 0.9–8.6%; Table 3). This is the first report of sexually originated progeny in facultative apomictic tetraploids in these species. 

### 2.4. Competition among Reproductive Pathways through the Reproductive Stages 

The level of apomixis and sexuality expression varied at each reproductive stage, but *P. cromyorhizon* and *P. maculosum* showed a similar pattern. Both species showed a gradual increase in the expression of apomixis at the cost of sexuality throughout the reproductive stages from the ovule to offspring, while maintaining a low degree of residual sexuality in the last stages.

**Figure 2:**
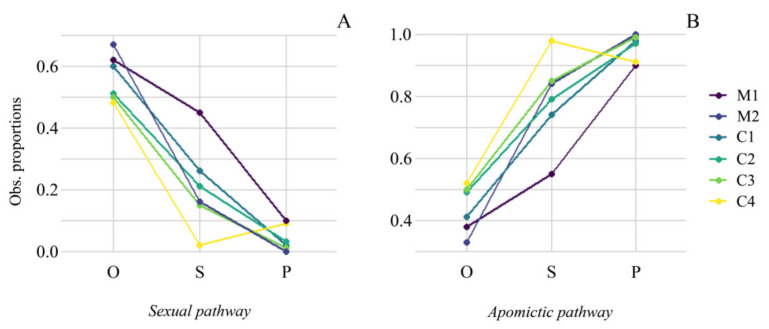
Observed proportions of the sexual (**A**) and apomictic (**B**) pathway in three reproductive stages: ovules (O), seeds (S), and progeny (P) in *P. maculosum* and *P. cromyorhizon*.

The proportion of sexuality was the highest at the ovule stage in both species (Table 1) compared to the seed (Table 2) or the progeny stages (Table 3, Figure 2). The progeny stage showed the lowest proportion of sexuality for these species. All populations showed significant differences between observed and expected proportions of sexually originated seeds (*p* < 0.001, Table 4). Significant differences were also found between observed and expected proportions of sexually originated progenies (*p* < 0.001, Table 5). These results showed a continuous reduction of sexuality along the reproductive stages during offspring formation (Figure 2). Nevertheless, such a reduction is not complete, and tetraploids in both species produced sexual offspring at low frequencies, both at seed and progeny stages (Figure 2). 

Regarding the efficiency of the sexual pathway, from the ovule to the seed stage, it ranged from 0.24–0.73 in *P. maculosum* and from 0.04–0.43 in *P. cromyorhizon* (Table 4). Values of reproductive efficiency lower than one represents a low efficiency of that reproductive pathway at that stage. Thus, the efficiency of the sexual pathway decreases from the ovule to the seed stage. Likewise, the efficiency of the sexual pathway in the seed to progeny stage ranged 0.0–0.22 in *P. maculosum* and 0.07–4.5 in *P. cromyorhizon* (Table 5). Therefore, the turn-over rate of the sexual pathway decreases from the seed to progeny stages, except for the population C4 of *P. cromyorhizon* (sexual reproductive efficiency: 4.5).

On the other hand, the proportion of apomixis was the highest at the progeny stage in both species (Table 3) compared to other stages (Table 1 and Table 2, Figure 2). All the populations showed significant differences between the observed and expected proportions of apomictic seeds (*p* < 0.001, Table 4). These differences were also noticed between the observed and expected proportions of clonal progenies (*p* < 0.001, Table 5).

The efficiency of the apomictic pathway from the ovule to seed stage ranged 1.45–2.55 in *P. maculosum* and 1.6–1.88 in *P. cromyorhizon* (Table 4). These values are higher than one, which indicates that the efficiency of this pathway increases from the ovule to the seed stage. The efficiency of the apomictic pathway from the seed to the progeny stage ranged from 1.19–1.64 in *P. maculosum* and from 0.93–1.32 in *P. cromyorhizon*, which also indicates an increased efficiency of apomixis (Table 5). In contrast, the apomictic efficiency of 0.93 in *P. cromyorhizon* population C4 indicates a slightly lower efficacy of apomixis, whereby the observed number of clonal progenies was lower than expected.

## 3. Discussion

Sexual and apomictic pathways in plants can coexist in the same individual at different rates [23,31,47]. In *P. cromyorhizon* and *P. maculosum*, the expression of sexuality decreased toward the final stages of reproduction but maintained low rates of residual sexuality at the tetraploid level. In contrast, the expression of apomixis showed an increase towards the final stages but never outcompeted sexuality.

### 3.1. Becoming a Seed: The Advantages of Aposporic Embryo Sacs

Under which circumstances is it to expect that two alternative reproductive pathways concurring in the ovule stage have the same chance to produce seeds? Even though the 4*x* of *P. cromyorhizon* and *P. maculosum* are facultative apomictic at the ovule stage, due to the simultaneous presence of both mature and fully developed meiotic and aposporic embryo sacs, the chances of them producing seeds are not the same. Both species showed a decrease in the expression of the sexual pathway from ovule to seed.

Competition between meiotic and aposporic embryo sacs shows a bias toward the apomictic pathway. Previous studies identified relevant factors influencing this competition in favor of the sexual or apomictic pathways. These factors can be gathered in numerous groups: polyploidy [2,9,23,27,48]; hybridization [2,48]; sexual pathway deregulation [14,49]; differential gene expression in the ovule [2,8,14,30,31,50]; inbreeding depression [31]; genetic disharmony in seminal tissues [51,52,53] (i.e., EBN, imprinting); light regime [11,23]; water stress [9,25]; environmental conditions [10,41]; seasonal variation of the flowering time [23,24]; pollination timing [28,54]; precocious development and parthenogenesis [21,30,31,48,55,56]. Studies suggest that a combination of all or some of these factors may be at play, affecting the reproductive pathways in the ovule [57].

The apomictic pathway is considered a “shortcut” or deregulation of the key processes of the sexual pathway, i.e., meiosis and fertilization [2,58]. This deregulation could be the product of heterochronicity or heterotopicity of the sexual processes caused by a perturbation or change in the genic expression such as polyploidy or hybridization, e.g., [2,18,22]. In aposporic species, like *P. maculosum* and *P. cromyorhizon*, nucellar cells can develop into aposporic megagametocytes (heterotopicity). These aposporic initials have a precocious development during megasporogenesis, and the embryos in these aposporic embryo sacs also have a precocious development through parthenogenesis (heterochronicity). This heterochronicity and heterotopicity combined can be an advantage for AES when they compete for space and resources within the ovule against MES. Differences at the beginning of the embryo development between MES and AES were first seen in *Calamagrostis* Adanson [59]. Savidan and Pernés [60] also saw a clear temporal difference in the development of apomictic and sexual pathways in *Panicum maximum* Jacq. At anthesis, most AES in apomictic plants of *P. maximum* were mature, while in sexual plants only 2/3 of the ovules showed a mature and complete MES.

Martínez et al. [54] observed that when a 4*x* apomictic of *Paspalum notatum* Flüggé is artificially pollinated during 2–3 days before anthesis, B_III_ hybrids (2*n* + *n*) can be obtained at low frequencies, showing that the development and maturation of AES takes place prior to the anthesis, and this probably allows these embryo sacs to avoid fertilization during anthesis. In many *Paspalum* species, parthenogenetic development plays a crucial role, increasing the reproductive efficiency of apomixis by allowing the precocious development of apomictic embryos. The presence of pro-embryos in AES has been repeatedly observed in ovules at anthesis and in absence of pollen in some *Paspalum* species [31,61,62,63,64,65]. The observation of pro-embryos in aposporic sacs at anthesis was 3% and 4.9% in *P. maculosum* and *P. cromyorhizon*, respectively. In these pseudogamous species, the endosperm development starts with pollination. As parthenogenesis allows the beginning of aposporic embryos development, at pollination the apomictic embryo growth is more advanced than the sexual one [31,54]. However, these values are not enough to explain the higher proportion of apomictic seeds.

Another possibility in the decline of sexual seed production could be due to an intrinsic factor that apomictic species in *Paspalum* produce a lower proportion of seed than their sexual counterparts [26,55,66,67,68]. However, the sources of this fertility loss are still unknown, so it cannot be attributed to the failure of the sexual pathway alone. Hojsgaard et al. [31] discussed the possibility that seeds obtained through open pollination probably came from self-pollinations. If so, sexual seeds would increase the number of genes at homozygosis. This leads to noxious effects caused by the inbreeding depression [69,70,71] which likely affects the normal development of the sexual seeds and leads to a decrease in the observed proportion of these seeds. The observed values of sexual seeds in the facultative apomictic tetraploids of *P. cromyorhizon* and *P. maculosum* are similar to those previously registered in others *Paspalum* species, e.g., [10,13,31,43]. Although there is a decrease in the production of sexual seeds, the observed values show a wide variation reaching up to 45% of total seeds in the M1 population of *P. maculosum*. This showed that the ability to produce variable progeny is retained at the seed stage and that there are genotypes in which the production of sexual and apomictic seeds can be almost 1:1. This value in apomictic tetraploids of the genus is between 0–30% of sexual seeds [10,31,43].

As discussed in previous studies [31,48,72], the relaxation in the relative contributions of maternal and paternal genomes to the development of the endosperm tissues observed in many pseudogamous apomicts, including tetraploids of *Paspalum*, can also explain the observed percentages of sexual seeds in *P. cromyorhizon* and *P. maculosum*. A DNA content ratio of 2:1 between maternal and paternal is one of the biggest limitations in the development of endosperm in sexual seeds [52,53]. Any deviation to this rule inhibits seed formation and it is one of the main causes of seed abortion in heteroploid or inter-specific hybridizations in sexual plants [51,73]. This limitation has not been observed in apomictic plants of *Paspalum*, in which seeds can be formed independently of the paternal progenitor ploidy [51]. Sexual seed production has also been noticed in other apomictic genera, such as *Taraxacum* F. H. Wigg. [74,75] and *Hieracium* L. [76]. The high proportion of sexual seeds could also explain the difficulty to obtain vigorous progeny if we consider that less adaptive combinations or chromosomic imbalances can result in the sexual pathway. Our results showed that there is a clear competitive advantage of apospory to produce seeds in *P. cromyorhizon* and *P. maculosum*. Similar to the relative advantages of aposporous versus meiotic female gametophyte developments found in other *Paspalum* species [31], our study provides similar lines of evidence. One of these advantages relates to the precocious and fast development of the apomictic pathway observed in different species and the genetic imbalance produced by sexual pathway in the embryo and endosperm of sexual seeds, which seem to be the most relevant factor influencing the functionality and competitiveness of each reproductive pathway in *P. cromyorhizon* and *P. maculosum*. However, this advantage is not enough to displace the sexual pathway at the seed stage. In facultative apomicts like observed in *P. cromyorhizon* and *P. maculosum*, the frequency of sexual and apomictic seeds may also be influenced by environmental conditions and local adaptation in apomictic populations [13]. The final output of this competition will directly shape the genetic contribution of each apomictic genotype to the gene pool of the population, which will consequently alter the allelic frequencies and genotypic diversity in the following generations.

### 3.2. Non-Clonal Progeny and Their Evolutive Role in Apomictic Populations

A maternal apomictic genotype is considered to be an obligate apomictic when all its offspring show a maternal genotype. Obligate apomictic genotypes might exist at variable frequencies in nature, as 100% clonal progeny has been reported in different species e.g., [31,77,78,79]. Furthermore, many studies showed that the sexual pathway is not completely blocked in apomictic species, and therefore sexual reproduction can still occur, at least occasionally. However, most approaches referred to residual sexuality as the proportion of sexual seeds produced by an apomictic plant, e.g., *Boechera holboelli* (Hornem.) Á. Löve and D. Löve [35], *Ranunculus kuepferi* Greuter and Burdet [34], or some *Paspalum* species [10,43], and only a few studies focused on the production of clonal progeny, e.g., *Bothriochloa–Dichantium* complex [32], *Rubus* L. [80], *Hieracium* sub-genus *Pilosella* [76], *Pilosella officinarum* Vaill. (ex *Hieracium pillosella* L.) [81], *Paspalum notatum* [24] and *P. cromyorhizon*, and *P. maculosum* (e.g., this work). For example, previous reports regarding rates of residual sexuality in progeny tests in apomictic polyploids ranged from 14–17% in two *Rubus* species [80], 0.6–21% in three *Dichantium* Willemet species, 6–10% in *Bothriochloa grahamani* (Haines) Bor [80], 4–5% in *Paspalum notatum* [24], ca. 3% in two *Hieracium* species [76] and 0.2–2.7% in *Pilosella officinarum* [81]. In the present study, tetraploids of *P. cromyorhizon* showed levels of residual sexuality ranging from 0.9–8.6%, and 0.0–10% in *P. maculosum*. These results might support the hypothesis that (epi)genetic deregulation leads to the unstable development of sexual seeds, which causes a lower efficiency of the sexual pathway in tetraploid apomictic plants [31,58]. As discussed above, in apomictic plants the effects of inbreeding depression are effective only when the progeny has a sexual origin. Lower growth rates and less vigor are consequences of inbreeding, both in allogamous and autogamous plants [70], and it could be a possible explanation for the lowest germination capacity and short viability of sexual seeds from apomictic species.

Although the decrease in the expression of the sexual pathway from the seed to the progeny stage was notorious in *P. cromyorhizon* and *P. maculosum*, low proportions of recombinant, genetically variable offspring were recovered in these species. This reproductive flexibility provides survival advantages under changing environmental conditions by combining reproductive assurance with sexuality. Rebozzio et al. [24] observed a decrease of the expected proportion of sexual progeny during the flowering peak in *Paspalum notatum*, considering the cytoembryological analyses from previous studies [82,83]. They also observed that this proportion increased towards the end of the flowering season [24]. This change in the expression of the reproductive pathway would allow the creation of variable progeny when environmental conditions are unfavorable and would maintain the production of clonal progeny when environmental conditions are auspicious [23,24]. Our analysis was performed in the peak of the flowering season of each species, which is in the middle of the spring season (astronomical season) in the southern hemisphere (*P. maculosum*: middle–end October; *P. cromyorhizon*: end October–beginning November, Reutemann pers. obs.). According to Quarin [23], tetraploids of *P. cromyorhizon* show the highest observed number of AES at the peak of the flowering season. Similar results were noticed in grasses of the Maximae complex (*Panicum maximum* Jacq., *P. infestum* Anders, and *P. trichocladum* K. Schum., Panicoideae), in which sexuality at the ovule stage varied in the ranged of 10–90%, depending on the environmental conditions [60,61,62,63,64,65,66,67,68,69,70,71,72,73,74,75,76,77,78,79,80,81,82,83,84]. Thus, at least in *P. cromyorhizon*, the levels of residual sexuality observed at the flowering peak where the expression of apomixis is expected to be at the highest provide good evidence that sexuality is functional in this species despite the possible competitive burden between the reproductive pathways.

The changes in the rates of sexuality or apomixis during offspring formation will affect the genotypic composition of natural populations in subsequent generations of facultative apomictic species. If apomictic rather than sexual offspring is preponderant, the genotypic (and genetic) variability in natural populations is expected to diminish, e.g., [85,86,87]. Nonetheless, apomixis (especially apospory) is not an obligated or irreversible condition, and it can be interrupted by sexual reproduction events that originate new genetic variants in the population e.g., [2,57,88,89], as seen in natural populations of *Pilosella officinarum* (see [90]) and in *P. cromyorhizon* and some *P. maculosum* populations (this work). Therefore, the production of non-clonal progeny by some plants within the population buffers the reduction in genotypic (and genetic) diversity expected within populations of apomicts, which in turn allows greater adaptability under different environmental conditions [13,91,92]. In addition, as few as 5% sexual events in apomictic polyploids prevent deleterious mutations to accumulate, avoiding genomic decay and the extinction of apomictic lineages [12,93]. Thus, the low levels of sexual offspring produced by *P. cromyorhizon* and *P. maculosum* apomictic genotypes are expected to have a central role in natural populations, securing adaptability through the exploitation and maintenance of extant genetic variability.

## 4. Materials and Methods

### 4.1. Plant Material

Tetraploid plants were randomly selected from three monoploid populations and one multiploid (2*x*–4*x*) population of *P. cromyorhizon*, and two multiploid (2*x*–4*x* and 2*x*–3*x*–4*x*) populations of *P. maculosum* [94]. All populations were collected in Corrientes Province, Argentina. Population sampling was done by collecting rhizome cuttings from single plants in their natural environment. Samples were taken at least 10 m apart from each other to avoid sampling the same individual genotype twice. The cuttings were grown in pots in a greenhouse which were then transferred to the experimental field of the Facultad de Ciencias Agrarias, Universidad Nacional del Nordeste, Corrientes, Argentina. Voucher herbarium specimens for each sampled population were collected in the field and deposited at MNES (Herbario de la Universidad Nacional de Misiones, Instituto de Biología Subtropical, UNaM-CONICET, Misiones, Argentina) (see Table S1 for information about ploidy level composition and sampling location). Five tetraploid plants within each population were selected to determine the reproductive mode at three developmental stages by using three different methodologies: cytoembryological analysis of megagametophytes in mature ovules, flow cytometry seed analysis, and progeny test using ISSR molecular markers.

### 4.2. Cytoembryological Analyses of Female Gametophytes

During peak flowering, spikelets at anthesis were collected and fixed in FAA (18:1:1, 70% ethanol: glacial acetic acid: formaldehyde) for 24 h, transferred to 70% ethanol, and storage at 4 °C. At least 30–35 individual florets were dissected using a Leica EZ4 stereomicroscope (Leica, Wetzlar, Germany). Pistils were cleared using the method described in Young et al. [95] with the modifications introduced by Zilli et al. [96]. Pistils were treated with 3% H_2_O_2_ for 2 h prior to dehydration in an ethanol series (50, 70, 95, and 100% steps; 30 min each step). Finally, dissected pistils were cleared using a series of methyl salicylate/ethanol (*v*/*v*) solutions (1:1, 3:1, 5.6:1; 30 min each step), incubated in methyl salicylate for at least 12 h, and examined using a Leica DM2500 (Leica, Wetzlar, Germany) microscope equipped with differential interference contrast (DIC) optics. Around 30–35 ovules per plant, 150–250 ovules per population, were analyzed and classified into sexual or asexual according to the observed types of embryo sacs. Ovules bearing an embryo sac with an egg apparatus carrying an egg-cell and two synergid cells at the micropyle, a large two-nucleate central cell, and several antipodal cells at the chalaza, were considered meiotic (MES) in origin. Alternatively, ovules bearing single or multiple embryo sacs lacking antipodal cells, differing in size and orientation, were recorded as aposporous (AES) in origin. In addition, ovules with both types of embryo sacs, i.e., ovules with mixed embryo sacs, were described as mixed ovules (MES + AES). Ovules without an embryo sac or with undeveloped or immature embryo sacs at anthesis were also counted (IES). We also estimated the percentage of apomictic pro-embryos observed, before the fertilization, in ovules bearing AES and MES + AES.

### 4.3. Flow Cytometry Seed Analysis

At the flowering peak, mature seeds were collected under open pollination conditions in each population. At least 30 seeds from each selected tetraploid were manually scarified to dissect out caryopses, which were rinsed in a series of 30% *v*/*v* hypochlorite, 70% *v*/*v* ethanol, and sterile distilled water (5 min each step). Nuclei were isolated and stained in two steps using Otto buffers [97]. First, nuclei were extracted by chopping caryopses into 0.5 mL extraction buffer Otto I for 30 s and filtered through a 30-μm nylon mesh (CellTrics^®^ Partec GmbH, Münster, Germany), followed by the addition of 1.5 mL of staining Buffer Otto II, which contains 4′,6′-diamidino- 2-phenylindole (DAPI). The DNA content (C value) was determined by measuring the fluorescence intensity of DAPI-stained nuclei using a CyFlow Space (Sysmex Partec, Goerlitz, Germany) flow cytometer in the blue fluorescence channel (UV LED, wavelength 365 nm). A tetraploid *P. cromyorhizon* or *P. maculosum* plant was used as the external reference to adjust the gain standard of the UV lamp for each species samples and the parameters were kept for all measurements. Bulks of two seeds were used and histograms were analyzed using the software FloMax version 2.8.1 (Quantum Analysis GmbH, Münster, Germany). A maximum coefficient of variation (CV) value of 5% was accepted for each sample peak. The relative fluorescence of at least 3000 particles (nuclei) was measured for each sample, and histogram peaks were assigned to embryo and endosperm tissues following the rationality described in [31]. The flow cytometry seed screen (FCSS) method allows the sexual seeds to be distinguished from the apomictic seeds by comparing the relative embryo and endosperm DNA content in seeds [98,99]. Assuming that the C-value refers to the entire nuclear DNA of a plant, *Paspalum* seeds that originated sexually show a 2C: 3C embryo: endosperm ratio (peak index of 1.5), whereas asexually formed seeds showed a 2C:5C or 2C:6C ratio, corresponding with an unreduced egg cell developing parthenogenetically into an embryo (2C), and the two unreduced polar nuclei (4C) fertilized by either reduced (1C) or unreduced (2C) pollen nuclei (pseudogamous endosperm), respectively (peak index ≥ 2.5).

### 4.4. Progeny Test and Molecular Profile Analyses

Mature seeds obtained at open pollination conditions at the flowering peak of each selected apomictic tetraploid were germinated under greenhouse conditions (24 °C, 10–12 h light). At least 20 seedlings of each apomictic genotype were analyzed. Total genomic DNA was isolated using 50 mg of young leaves from the maternal apomictic genotype and its progeny. Leaves were macerated with the help of a plastic fuse drill and 700 μL of extraction buffer cetyl-trimethylammonium bromide (CTAB) 2% (1 M Tris-HCl pH 7.5; 0.5 M ethylenediaminetetraacetic acid [EDTA] pH 8; 5 M NaCl; 1% β-mercaptoethanol) in a tube of 1.5 mL. The samples were incubated at 60 °C for 30 min. Then, 700 μL chloroform was added and the mixture was stirred for 5 min and then centrifuged for 10 min. The aqueous phase was recovered and transferred to another tube. The nucleic acids were precipitated with 500 μL of cold 2-propanol. The tubes were then kept in a freezer at −20 °C for approximately 30 min. Then, the samples were centrifuged at 4 °C for 20 min. The supernatant was discarded, and the pellet was washed with a washing solution (70° EtOH + 0.2 M NaOAc) and centrifuged again for 10 min. After centrifugation, the supernatant was discarded again, and the pellet was suspended in 25 μL of sterile, tris-ethylenediaminetetraacetic acid (TE) buffer (1 M Tris-HC1 pH 8; 0.5 M EDTA pH 8) and kept in a refrigerator. The genomic DNA was quantified by visual comparison to a known patron by electrophoresis in 1% agarose gels in 1X TAE buffer (40 mM Tris-HCl; 5 mM NaOAc; 0.77 mM EDTA; pH 8.0) at 40 V for 1 h. Genomic DNA was visualized under ultraviolet (UV) light and photographed with GelDoc-It Imaging System (UVP LLC), after staining with ethidium bromide (1 µg mL^−1^). Each DNA sample was adjusted to 20 ng μL^−1^ for its use in polymerase chain reaction (PCR) amplification. A total of 19 ISSR primers were used for PCR amplification. The primers analyzed were: (AC)_8_G, (AC)_8_T, (AG)_8_C, (AG)_8_T, (AGAC)_4_GC, (AG)_8_GC, (ATG)_5_GA, (CA)_8_G, (CA)_8_T, CAG(CA)_7_, (CT)_8_G, (GA)_8_C, (GA)_8_G, (GA)_8_T, (GA)_8_TC, GAG(AC)_7_, (GT)_8_C, (GT)_8_TC, and (TC)_8_A. Reactions were performed in 25 mL final volume containing 20 ng of template DNA; 2.5 mL of reaction buffer 10X·; 2.5 mL of MgCl2 (50 mM); 1.0 mL of primer (10 pmol mL^−1^); 1 mL of dNTP (10 mM); 0.2 mL of Taq DNA polymerase (5 U ml^−1^) and ultrapure H2O to complete 25 mL. DNA amplifications were done in a T100 thermal cycler (Bio-Rad) with the following thermal cycle: initial denaturing at 94 °C for 5 min; 40 cycles of 94 °C for 1 min, 46–55 °C for 45 s (depending on the primer), 72 °C for 2 min, and a final extension at 72 °C for 5 min. Polymerase chain reaction products were separated by electrophoresis in 2% agarose gels in 1X TAE buffer at 70 V for 4 h and stained with ethidium bromide (1 µg mL^−1^). The molecular profiles were visualized under UV light, photographed, and stored for further analysis with GelDoc-It Imaging System. The number of genotypes (nG) in the progeny was determined using the GenoType and GenoDive Software [100] under the infinite alleles model and using different number of mutational steps (S). We considered three mutational steps S = 3 for the analyses to avoid possible genotyping or amplification errors but extended the analysis from S = 0 to 15. Progeny were considered to have an apomictic origin when they showed an identical molecular profile to their maternal apomictic genotype. Progeny were considered to have a sexual origin when they showed a non-identical (polymorphic) molecular profile compared to their maternal apomictic genotype. We determined the number of progeny that had the maternal genotype (i.e., clonal) as C and the number of progeny showing a genotype different to their maternal apomictic genotype (i.e., non-clonal) as nC.

### 4.5. Reproductive Pathway Efficiency Assessment

The efficiency of each reproductive pathway (sexual and apomictic) was calculated as the ratio between the observed and the expected proportions of flowers undergoing the meiotic or apomictic pathway [10,31]. The reproductive efficiency is one when both pathways have the same effective chance of continuing to the next reproductive stage, is less than one when they have a less chance to continue, and more than one when they have a bigger chance.

The observed proportion of embryo sacs was estimated as *nm*/*nt* for the meiotic pathway and *na*/*nt* for the apomictic pathway. Here, *nm* is the total number of ovules with a meiotic embryo sac (MES), and *na* is the total number of ovules with apomictic embryo sacs (AES), where both *nm* and *na* include the number of observed ovules with both meiotic and apomictic pathways (MES + AES), and *nt* is the total number of embryo sacs. The number of sexual and apomictic seeds was used to calculate the observed proportions of each reproductive pathway at the seed stage, and the expected proportion of sexual and apomictic seeds were the observed proportions at the ovule stage. The number of genotypically non-clonal (nC) and clonal (C) progeny was used to calculate the observed proportions of the sexual and apomictic pathway at the progeny stage, respectively. The expected proportions of sexual and apomictic progeny were equal to the observed proportions at the seed stage. In our analysis, it was assumed that (i) MES and AES have independent development from each other, (ii) and the same chance of successfully growing into a seed, (iii) sexual and apomictic seeds have the same probability of successfully originate a new individual for the next generation. A paired *t*-test was performed on the mean difference between the observed proportions of both pathways in each stage. Besides, a standard Pearson’s Chi-squared test was performed to check for significant differences between observed and expected proportions of each pathway when going through one stage to the next (ovule–seed and seed–progeny). Both tests were performed using R software [101]. Briefly, we examined the presence of meiotic and/or apomictic embryo sacs in the ovules, which of these embryo sacs successfully developed into a seed, and whether these seeds originate a clonal or non-clonal new generation (Figure S1).

## 5. Conclusions

This work has demonstrated that the expression of apomixis and residual sexuality changes through the reproductive development stages in apomictic *Paspalum* species. The outcome of these two potential reproductive pathways, analyzed from the embryo sacs formation through the survival of the progeny, impacts not only the genetic diversity of future generations but also population dynamics regarding reproduction structure. Additionally, it is important to include not only the potential of sexuality or apomixis in ovules or seed analysis in future analysis regarding apomixis, but also the realized rate of sexuality and apomixis in the next generations, as we noted that it could change from one stage to the next. A facultative apomictic plant could have potential for sexuality at the ovule or seed stages, but in its filial, only those derived from the apomictic pathway survive, leading to different consequences in population dynamics and also in agronomical approaches that validate themselves using apomixis technology.

## Figures and Tables

**Table 1:** Observed number and percentage (%) of embryo sac types in tetraploids of *P. maculosum* and *P. cromyorhizon*, and the observed proportions of the sexual (SP) and apomictic (AP) pathways at ovule stage.

Species	Pop	*n*	Number of Ovules Bearing (%)				Proportions		
			MES	AES	MES + AES	IES	SP	AP	*p*
*P. maculosum*	M1	146	51 (34.9)	-	81 (55.5)	14 (9.6)	0.62	0.38	<0.001
	M2	179	83 (46.4)	3 (1.7)	72 (40.2)	21 (11.7)	0.67	0.33	<0.001
*P. cromyorhizon*	C1	60	19 (31.7)	5 (8.3)	25 (41.7)	11 (18.3)	0.59	0.41	0.13
	C2	160	8 (5.0)	3 (1.9)	144 (90.0)	5 (3.1)	0.51	0.49	0.82
	C3	150	4 (2.7)	6 (4.0)	138 (92.0)	2 (1.3)	0.50	0.50	0.95
	C4	148	8 (5.4)	17 (11.5)	119 (80.4)	4 (2.7)	0.48	0.52	0.62

*n*: total number of analyzed ovules. MES: meiotic embryo sac, AES: aposporic embryo sac, MES + AES: meiotic embryo sac plus one or more aposporic embryo sacs, IES: immature or undeveloped embryo sacs; significant differences (*p* < 0.05).

**Table 2:** Observed number and percentages (%) of seeds with different C-DNA ratios of embryo: endosperm in tetraploids of *P. maculosum* and *P. cromyorhizon*, and the observed proportions of the sexual (SP) and apomictic (AP) pathway at the seed stage.

Species	Pop	*n*	*n* (%) Seeds with			Proportions		
			2C:3C	2C:5C	2C:6C	SP	AP	*p*
*P. maculosum*	M1	152	68 (44.7)	84 (55.3)	-	0.45	0.55	0.22
	M2	120	19 (15.8)	101 (84.2)	-	0.16	0.84	<0.001
*P. cromyorhizon*	C1	62	16 (25.8)	46 (74.2)	-	0.26	0.74	<0.001
	C2	162	34 (21.0)	126 (77.8)	2 (1.2)	0.21	0.79	<0.001
	C3	154	23 (14.9)	130 (84.4)	1 (0.7)	0.15	0.85	<0.001
	C4	140	3 (2.1)	137 (97.9)	-	0.02	0.98	<0.001

*n*: number of analyzed seeds, 2C: 3C, C-DNA ratio corresponding to a meiotic seed; 2C: 5C, C-DNA ratio corresponding to an apomictic seed; 2C: 6C, C-DNA ratio corresponding to an apomictic seed with polar nuclei fertilization with an unreduced pollen grain; significant differences (*p* < 0.05).

**Table 3:** Observed number and percentages (%) of progenies genotypically clonal (C) and non-clonal (nC) to their apomictic genotype progenitor in tetraploid *P. maculosum* and *P. cromyorhizon* with three mutational steps (S = 3), and the observed proportions of the sexual (SP) and apomictic (AP) pathway at the progeny stage.

Species	Pop	*n*	*n* (%) of Progeny		Proportions		
			C	nC	SP	AP	*p*
*P. maculosum*	M1	60	54 (90.0)	6 (10.0)	0.10	0.90	<0.001
	M2	52	52 (100)	0 (0.0)	0.00	1.00	<0.001
*P. cromyorhizon*	C1	46	45 (97.8)	1 (2.2)	0.02	0.98	<0.001
	C2	106	103 (97.2)	3 (2.8)	0.03	0.97	<0.001
	C3	105	104 (99.1)	1 (0.9)	0.01	0.99	<0.001
	C4	105	96 (91.4)	9 (8.6)	0.09	0.91	<0.001

*n*: total number of analyzed progeny plants. Significant differences (*p* < 0.05).

**Table 4:** Expected (Ei) and observed (Oi) proportions of sexuality and apomixis at the seed stage and the reproductive efficiency of each pathway.

Species	Pop	Proportions				χ^2^	*p*	Reproductive Efficiency	
		Sexual Pathway		Apomictic Pathway					
		Ei	Oi	Ei	Oi			Sex	Apo
*P. maculosum*	M1	0.62	0.45	0.38	0.55	10.0	<0.001	0.73	1.45
	M2	0.67	0.16	0.33	0.84	81.8	<0.001	0.24	2.55
*P. cromyorhizon*	C1	0.59	0.26	0.41	0.74	12.5	<0.001	0.43	1.8
	C2	0.51	0.21	0.49	0.79	37.7	<0.001	0.42	1.61
	C3	0.50	0.15	0.50	0.85	50.0	<0.001	0.3	1.7
	C4	0.48	0.02	0.52	0.98	86.9	<0.001	0.04	1.88

χ^2^, Chi-square statistics, significant differences (*p* < 0.05).

**Table 5:** Expected (Ei) and observed (Oi) proportions of sexuality and apomixis at the progeny stage and the reproductive efficiency of each pathway.

Species	Pop	Proportions				χ^2^	*p*	Reproductive Efficiency	
		Sexual Pathway		Apomictic Pathway					
		Ei	Oi	Ei	Oi			Sex	Apo
*P. maculosum*	M1	0.45	0.10	0.55	0.90	21.3	<0.001	0.22	1.64
	M2	0.16	0.00	0.84	1.00	7.7	<0.001	0.0	1.19
*P. cromyorhizon*	C1	0.26	0.02	0.74	0.98	9.4	<0.001	0.08	1.32
	C2	0.21	0.03	0.79	0.97	16.3	<0.001	0.14	1.23
	C3	0.15	0.01	0.85	0.99	12.9	<0.001	0.07	1.16
	C4	0.02	0.09	0.98	0.91	4.0	<0.001	4.5	0.93

χ^2^, Chi-square statistics, significant differences (*p* < 0.05).

## Data Availability

ISSR raw data are available on request from the corresponding authors. All the data presented in this study are available in the article and in Supplementary Materials.

## Supplementary Materials

The following supporting information can be downloaded at: https://www.mdpi.com/article/10.3390/plants11131639/s1, Figure S1. Boxplot of the observed number of ovules (O), seeds (S) and progenies (P) in each reproductive pathway (sexual and apomictic) in (A) *P. maculosum* and (B) *P. cromyorhizon*, Table S1. Ploidy level composition (2n), sampling location of populations of *P. maculosum* and *P. cromyorhizon*, voucher and herbarium where deposited. Table S2. Percentages of polymorphic bands (% PL) in the analysis of each maternal apomictic genotype and it progeny considering no mutational steps (S = 0) and three mutational steps (S = 3). Table S3. Number of non-clonal genotypes (NCG) in the progeny of each maternal apomictic genotype considering three mutational steps (S = 3).
