# Supplementary material for: Variation of Residual Sexuality Rates along Reproductive Development in Apomictic Tetraploids of Paspalum
Source: Plants (Basel). 2022 Jun 21;11(13):1639. doi: 10.3390/plants11131639 (PMC9269205; doi:10.3390/plants11131639)
Supplement: Supplementary file 1 [file plants-11-01639-s001.zip › plants-1779457-supplementary.pdf]

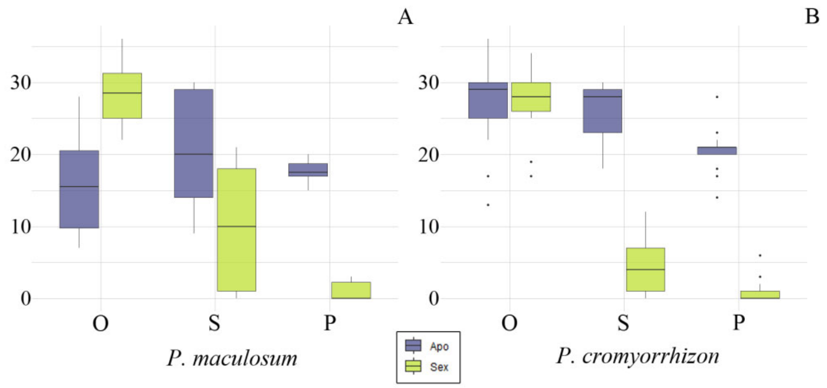

Figure S1. Boxplot of the observed number of ovules (O), seeds (S) and progenies (P) in each reproductive pathway (sexual and apomictic) in (**A**) *P. maculosum* and (**B**) *P. cromyorrhizon*.

Table S1. Ploidy level composition (2*n*), sampling location of populations of *P. maculosum* and *P. cromyorhizon*, voucher and herbarium where deposited.

Species	Pop	2*n*	Location	Voucher and Herbarium
P. maculosum	M1	20,30,40	S 28.34389; W 55.93099	*Honfi 2145*, CTES, MNES
	M2	20,40	S 28.67125; W 56.180694	*Honfi 2232*, CTES, MNES
P. cromyorhizon	C1	20,40	S 29.55815; W 57.50227	*Honfi 1732*, CTES, MNES
	C2	40	S 29.84539; W 57.66850	*Honfi 1733*, CTES, MNES
	C3	40	S 29.95637; W 58.81866.	*Honfi 1735*, CTES, MNES
	C4	40	S 30.628889; W 57.983861	*Honfi 1955*, CTES, MNES

Table S2. Percentages of polymorphic bands (% PL) in the analysis of each maternal apomictic genotype and it progeny considering no mutational steps (S= 0) and three mutational steps (S= 3).

Species	Apomictic Genotype	% PL	
		S= 0	S= 3
P. maculosum	M1 #1	6.4	0.0
	M1 #8	38.7	34.5
	M1 #9	22.7	19.3
	M2 #4	2.6	0.0
	M2 #5	0.0	0.0
	M2 #7	1.9	0.0
P. cromyorhizon	C1 #4	7.2	7.2
	C1 #20	0.0	0.0
	C2 #5	0.0	0.0
	C2 #6	7.2	2.2
	C2 #15	2.8	0.0
	C2 #17	6.1	0.0
	C2 #20	17.1	14.9
	C3 #4	3.3	0.0
	C3 #10	2.2	2.2
	C3 #11	5.0	0.0

C3 #15	0.6	0.0
C3 #19	1.1	0.0
C4 #3	0.0	0.0
C4 #8	12.7	12.2
C4 #12	1.7	0.0
C4 #16	13.3	13.3
C4 #20	5.5	0.0

Table S3. Number of non-clonal genotypes (NCG) in the progeny of each maternal apomictic genotype considering three mutational steps (S= 3).

Species	Maternal Apomictic Genotype	N	NCG
P. maculosum	M1 #1	20	0
	M1 #8	20	2
	M1 #9	20	3
	M2 #4	20	0
	M2 #5	14	0
	M2 #7	18	0
P. cromyorhizon	C1 #4	30	1
	C1 #20	17	0
	C2 #5	22	0
	C2 #6	22	1
	C2 #15	21	0
	C2 #17	21	0
	C2 #20	20	2
	C3 #4	21	0
	C3 #10	21	1
	C3 #11	21	0
	C3 #15	21	0
	C3 #19	21	0
	C4 #3	21	0
	C4 #8	20	3
	C4 #12	23	0
	C4 #16	20	2
	C4 #20	21	0

N, total number of descendants; *NCG*, number of genotypes different from the maternal apomictic genotype.
